# Ultrafast Hot‐Carrier Cooling in CsPbBr_3_ Supercrystals via Long‐Range Electronic Coupling

**DOI:** 10.1002/advs.75913

**Published:** 2026-06-09

**Authors:** Junhong Yu, Manoj Sharma, Yadong Han, Ke Wang, Zhenzhong Lian, Chang Cao, Lan Nguyen, Naufan Nurrosyid, Baiquan Liu, Jacek J. Jasieniak

**Affiliations:** ^1^ College of Physics and Electronic Engineering Chongqing Normal University Chongqing China; ^2^ Department of Materials Science and Engineering Clayton Campus Monash University Melbourne Victoria Australia; ^3^ School of Electronics and Information Technology Sun Yat‐sen University Guangzhou China

**Keywords:** ultrafast laser spectroscopy, delocalized electron, materials science, phonon, photovoltaics, perovskite, optoelectronics, spectroscopy, excitation, auger effect

## Abstract

Lead halide perovskite (LHP) nanocrystals (NCs) exhibit prolonged hot carrier (HC) cooling, which benefits photovoltaics but hinders light‐emitting applications. Current strategies to modulate HC dynamics often compromise intrinsic material properties or introduce competing photophysical processes. Here, we demonstrate that long‐range electronic coupling in CsPbBr_3_ supercrystals (SCs) efficiently accelerates HC cooling across different excitation regimes, enabling cooling dynamics approaching the prediction of the longitudinal optical phonon model. Owing to enhanced electronic density of states arising from cooperative interactions, transient absorption (TA) spectroscopy reveals SCs enable HC cooling that is twice as fast as isolated nanocrystals (NCs) at low carrier densities (2 × 10^17^ cm^−3^). Remarkably, under high excitation densities (4.3 × 10^18^ cm^−3^), carrier delocalization within the superlattices further weakens the spatial confinement‐induced Auger heating, accelerating HC cooling by over an order of magnitude. This work establishes SCs as a fresh platform to manipulate HC cooling.

## Graphical abstract

CsPbBr_3_ supercrystal self‐assembly, which induces long‐range electronic coupling, enables spatial carrier delocalization that accelerates carrier‐LO phonon scattering and weakens the Auger heating. These effects enable ultrafast, excitation‐intensity‐independent hot‐carrier cooling, offering a trade‐off‐free strategy for high‐performance perovskite‐based optoelectronics.

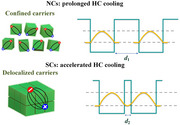

## 1 Introduction

Lead halide perovskites (LHPs) nanocrystals, owing to their remarkable defect tolerance, high radiative emission efficiency, superb charge mobility, and tunable band gaps [1, 2, 3], have recently emerged as promising low‐cost high‐performance materials for solar cells [4], light‐emitting diodes (LEDs) [5], and lasers [6]. These optoelectronic applications fundamentally rely on photon energy conversion (e.g., absorption or emission), which involves a complex interplay among different photophysical processes. As the first step of multistage photophysical phenomena, hot carrier (HC) cooling, where photoexcited carriers dissipate excess energy through phonon emission, exerts a pivotal influence on the performance of optoelectronic devices [7, 8]. Specifically, HC energy loss constitutes the primary efficiency bottleneck in photovoltaics, with a retarded cooling rate being considered one pathway to boost the solar collection efficiencies of single‐junction solar cells beyond the Shockley–Queisser limit [9]. In contrast, light‐emitting applications demand accelerated HC cooling to mitigate dynamic competition between intraband relaxation pathways and high‐energy‐level charge trapping, thereby ensuring efficient interband transitions [10, 11].

Compared to conventional organic [12], II–VI [13], and III–V [14] group semiconductors with HC cooling timescales of ∼0.1 ps, experimental evidence has consistently shown that LHP nanocrystals exhibit markedly slower HC cooling dynamics (> 0.4 ps) under moderate carrier densities [15, 16] due to polaronic screenings and intrinsic phonon bottleneck. Notably, the HC cooling timescale can be further extended by over an order of magnitude through hot phonon bottlenecks [17, 18] or Auger heating effects [9, 19] under higher carrier densities (e.g., > 10^18^ cm^−3^). Despite the prolonged HC lifetime highlighting the potential of LHP nanocrystals for efficient energy harvesting in photovoltaic applications, their performance in LEDs and lasers may be hindered. Accordingly, this dichotomy underscores the need for engineering HC cooling in LHPs, and several strategies, including the A‐site cation [20] or halide anion [21] change and the hetero‐interface formation [22], have successfully accelerated cooling rates in LHPs. However, these strategies will either tailor intrinsic material characteristics of LHPs [1, 3] or introduce additional photophysical processes (e.g., interface charge transfer) [23, 24].

In this work, we have shown that LHP supercrystals (SCs) with long‐range electronic couplings [25, 26] could accelerate HC cooling both at low and high excitation intensities. Using CsPbBr_3_ as a representative example, transient absorption (TA) spectra at the carrier density (*n*) of 2.1 × 10^17^ cm^−3^ or average generated electron–hole pairs (<*N*_0_>) of ∼0.21 without the influence of either hot‐phonon bottleneck or Auger heating effect, reveal a nearly two‐time faster HC cooling in SCs compared to that in NCs. This is attributed to the increased electronic density of states due to the cooperative interaction among individual NC states. Notably, the Auger heating effect becomes negligible in CsPbBr_3_ SCs with the carrier density up to ∼4.3 × 10^18^ cm^−3^ (i.e., <*N*_0_> of ∼4.6), leading to more than one order of magnitude faster HC cooling time compared to NCs in a similar excitation condition. The fluence‐dependent Auger dynamics confirm that electronic coupling among individual NCs within the superlattice results in the carrier delocalization, which thus weakens the efficient carrier–carrier interactions caused by spatial confinement in NCs.

## 2 Results

**FIGURE 1:**
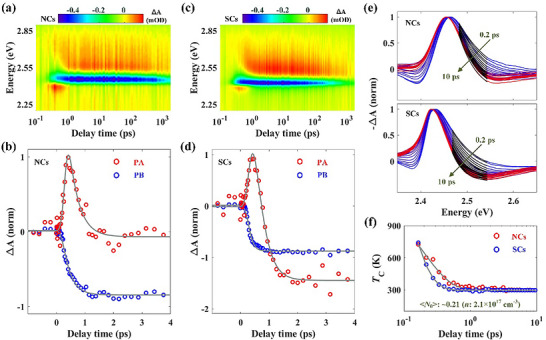
HC cooling in LHP NCs and SCs with low excitation densities (*n* = ∼2.1 × 10^17^ cm^−3^) and a photoexcitation energy of 3.1 eV. (a) Pseudocolor TA spectroscopies of CsPbBr_3_ NCs. (b) Early time TA dynamics of NCs probed at the positions of PA (red) and (blue) features. Silver lines are exponential fittings. (c) Pseudocolor TA spectroscopies of CsPbBr_3_ SCs. (d) Early time TA dynamics of SCs probed at the positions of PA (red) and PB (blue) features. Silver lines are exponential fittings. (e) Normalized TA spectra of NCs (top) and SCs (bottom) with different time delays. The black curves mark the data used for extracting the carrier temperature. (f) Hot‐carrier temperature of NCs (red) and SCs (blue) as a function of delay time.

The synthesis of colloidal cubic‐phase CsPbBr_3_ NCs and their in situ growth into SCs via hydrophobic interactions among hexadecylamine (HDA) ligand chains was recently reported by our group [27, 28]. The transmission electron microscopy (TEM) images of SCs in Figure S1 reveal a NC size of ∼10 nm in the intermediate‐confined regime and an inter‐NC distance of ∼2 nm. The scanning electron microscopy (SEM) image of SC films in Figure S2 clearly indicates the giant cubic morphology. The PL and absorption spectra of NCs and SCs are shown in Figure S3. Compared to isolated NCs, SCs exhibit a stronger Rayleigh scattering, a smaller Stokes shift, and a lower degree of quantum confinement, consistent with the formation of SCs with long‐range electronic couplings [29, 30]. We first investigate the TA dynamics at the low excitation density regime where the HC relaxation is not influenced by the hot‐phonon bottleneck or Auger heating effect. Figure 1 shows the TA spectra of CsPbBr_3_ NCs and SCs excited at ∼3.1 eV (400 nm) with average generated electron–hole pairs of ∼0.21 (<*N*_0_> = *J* × *σ*, where *J* is the pump fluence, and *σ* is the absorption cross‐section), which corresponds to the average carrier density of ∼2.1 × 10^17^ cm^−3^ (*n* = <*N*_0_>/*V*_NC_, where *V*_NC_ is the NC volume) [31].

For both NC and SC samples, pseudo‐color TA maps in Figure 1a,c show a negative photoinduced bleaching (PB) signal located around the bandgap, a short‐lived positive photoinduced absorption (PA) signal below the bandgap, and a long‐lived positive high‐energy tail above the bandgap, consistent with other studies of HC relaxation dynamics in LHPs [15, 16, 17, 18, 20, 21, 22, 32]. Since the positive PA signal is replaced by the PB feature when HCs arrive at the band edge during the initial thermalization and relaxation [20, 21], we can determine the time constant of the HC cooling process by comparing the PA decay and PB buildup. Accordingly, several key points can be concluded from Figure 1b,d: (i) the similar timescale of PA decay and PB buildup in both samples validates the HC relaxation and excludes the significant contribution of polaron formation in the early time excited state dynamics [33]; (ii) the HC cooling time is extremely fast (sub‐ps time scale) with low pump excitations, matching the intrinsic HC relaxation scenario [16]; and (iii) the HC cooling time in SCs has been observed to be nearly two‐time faster than that in NCs (211±9 fs versus 434±5 fs). To further confirm the intrinsic HC cooling difference in NCs and SCs, we have extracted the carrier temperature (*T*_C_) via fitting the high‐energy tail of the TA spectra with the Maxwell‐Boltzmann distribution function exp[(*E*_f_‐*E*)/*k*_B_ *T*_C_], where *k*_B_ is the Boltzmann constant, and *E*_f_ is the quasi‐Fermi energy. The normalized TA spectra at different time delays and corresponding fits of the high‐energy tails (i.e., the black curves) are presented in Figure 1e (also see the non‐normalized TA spectra of NCs and SCs in Figures S4 and S5, respectively).

It is well‐established that the effective masses of the electron and hole in LHPs are comparable [11, 15, 17], and *T*_C_ indicates an average temperature of the electrons and holes, which therefore can represent the cooling process of HCs. The temporal evolution of *T*_C_ for NCs and SCs with *n* = ∼2.1 × 10^17^ cm^−3^ is presented in Figure 1f. Both samples reach the maximum *T*_C_ of ∼730 K at ∼100 fs with low‐intensity excitation, confirming the similar excessive HC energy and initial HC densities at the excitation onset. Consistent with the dynamic analysis in Figure 1b,d, an obvious shortening of *T*_C_ relaxations occurred in SCs (i.e., nearly two times acceleration from ∼639 to ∼278 fs). Considering that both samples only have one electron and hole (i.e., <*N*_0_> of ∼0.21), many‐body effects are unlikely to explain the observed HC cooling difference. Many studies have reported that the intrinsic HC cooling rate (*k*_cool_) via multiphonon emission is related to several microscopic parameters of the system, including the overlap of electronic states with phonon modes, the phonon energy, the deformation potential, and the spring constant [21, 34]. Since CsPbBr_3_ NCs and SCs have the same lattice characteristics [35, 36], we follow the argument that efficient electronic coupling among individual NCs within the superlattice enhances the overlap integral of the electronic states with phonon modes, which thus accelerates the HC‐phonon scattering process. This argument can be further tested by the LO‐phonon model [15] (the gray curves in Figure 1f). *T*_C_ dynamics of NCs and SCs can be well‐reproduced in the absence of any hot‐phonon effects but with different LO phonon lifetimes (i.e., ∼176 fs in NCs and ∼64 fs in SCs), supporting the enhanced LO‐phonon scattering in SCs due to long‐range electronic couplings (also see the Raman spectra in Figure S11).

**FIGURE 2:**
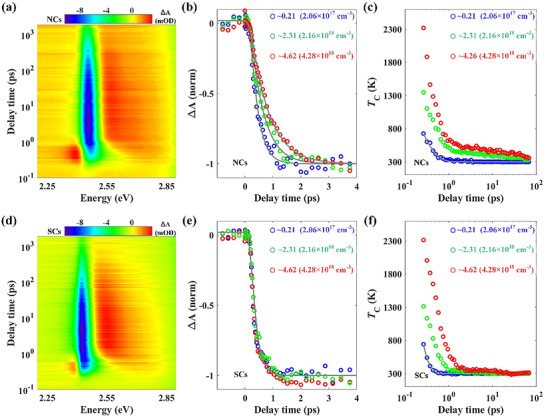
HC cooling in LHP NCs and SCs with different excitation densities. The photoexcitation energy is 3.1 eV. (a) Pseudocolor TA spectroscopies of CsPbBr_3_ NCs with *n* = ∼4.62 × 10^18^ cm^−3^. (b) The PB buildup dynamics in NCs with different excitation densities. (c) Temporal evaluation of *T*_C_ in NCs with different excitation densities. (d) Pseudocolor TA spectroscopies of CsPbBr_3_ SCs with *n* = ∼4.62 × 10^18^ cm^−3^. (e) The PB buildup dynamics in SCs with different excitation densities. (f) Temporal evaluation of *T*_C_ in NCs with different excitation densities.

Since HC cooling is governed by the intrinsic properties of materials, as well as extrinsic effects, such as multi‐particle Auger‐recombination and hot phonon bottlenecks, we next investigate HC dynamics in CsPbBr_3_ SCs at different carrier densities. Figure 2a shows a pseudocolor image of TA spectra for NCs with a carrier density of 4.28 × 10^18^ cm^−3^ (<*N*_0_> of ∼4.62). The pseudocolor images of TA spectra for NCs and SCs with a carrier density of 2.31 × 10^18^ cm^−3^ are provided in Figure S6. Similar to the analysis in Figure 1, the PB buildup dynamic and the transient relaxation of *T*_C_ have been utilized to indicate the HC lifetime in NCs (see the representative fits of high‐energy tails for NCs with different carrier densities in Figure S7). It is found that for NCs, both the decay time of *T*_C_ and the rise time of PB signals strongly depend on the carrier density: the rise time of PB signals can be prolonged to be larger than 1 ps, and an additional slow relaxation component in *T*_C_ dynamics even retards carrier temperature back to ambient conditions after tens of picoseconds. Such slow HC cooling in CsPbBr_3_ NCs at high carrier density has been identified in both all‐inorganic [21] and hybrid organic [20, 22] LHPs, in which the prolonged PB rising dynamics is mainly caused by the hot phonon bottleneck (i.e., accumulation of hot LO phonons with an inefficient Klemens channel) [17] and the emerged slow relaxation channel in *T*_C_ is attributed to the occurrence of Auger heating effects (i.e., carriers reheated by the interband Auger process) [9, 37].

As a comparison, the pseudo‐color TA map of SCs with a carrier density of 4.28 × 10^18^ cm^−3^ is shown in Figure 2d (see the analysis of TA spectra for SCs in Figure S8). It is noteworthy that the ultrafast PB buildup dynamics of ∼0.2 ps remain almost identical with varying carrier densities (see Figure 2e), even at a very high <*N*_0_> of 4.62. This suggests suppressed hot phonon bottlenecks in SCs. According to the classical LO‐phonon model, hot phonon bottlenecks occur in LHPs due to the large energy difference between LO‐ and longitudinal acoustic (LA)‐phonon branches, which severely hinders the Klemens‐type decay rate [15, 38]. As recently demonstrated by Sun et al. [39]. and Diroll et al. [40], closely packed nanoparticle superlattices with long‐range order could enable an interfacial Klemens channel for the LO‐phonon decay (i.e., the coupling of inter‐particle LO‐phonons with out‐of‐plane LA‐phonons), thus breaking the hot phonon bottleneck. Notably, the *T*_C_ dynamics shown in Figure 2f display a negligible contribution of the additional slow relaxation component, indicating that Auger re‐excitation, which serves as the main mechanism to decelerate HC cooling processes at high carrier densities [9], has also been greatly suppressed in SCs. This is particularly interesting for manipulating the excess energy of HCs since Auger heating is detrimental to both photovoltaic and light‐emitting applications (i.e., although Auger heating can prolong the HC cooling, it conversely consumes carrier densities) [16], which thus becomes the focus of this work.

**FIGURE 3:**
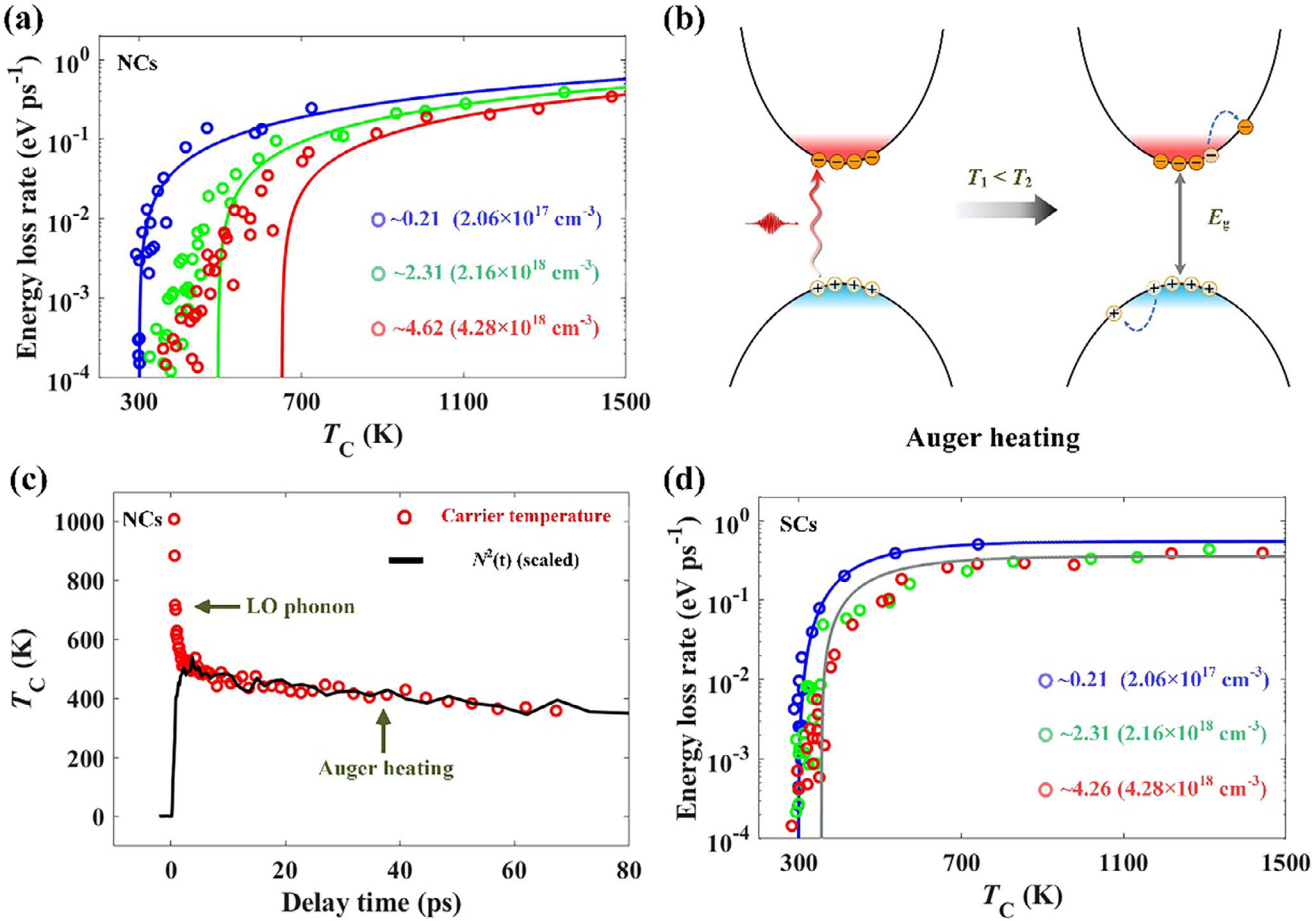
Analysis of the energy loss rate of HCs. (a) Energy loss rates as a function of electronic temperature for CsPbBr_3_ NCs. (b) Schematic illustrations of HC re‐excitation by non‐radiative Auger recombinations. *E*_g_: the energy bandgap. (c) Time‐resolved *T*_C_ compared with the square of the average number of electron–hole pairs, which is scaled to make two curves overlap at long time delays. (d) Energy loss rates as a function of electronic temperature for CsPbBr_3_ SCs.

To further confirm the mitigation of Auger heating effects in CsPbBr_3_ SCs, we take a closer look at the energy loss rate of HCs (*J*_r_), which could be inferred from the decay rate of *T*_C_ based on the following equation [41]: *J*_r_ = −1.5*k*_B_ × d*T*_C_/d*t*. As shown in Figure 3a, the initial energy loss rates of NCs for different <*N*_0_> are similar (i.e., around 0.2 eV ps^−1^), suggesting that carrier‐phonon interactions, which are insensitive to carrier densities [9, 15], dominate the onset of HC cooling. This argument is also supported by fitting the temperature‐dependent *J*_r_ using the carrier‐phonon coupling model [15, 22, 42]:

(1) $$J_{r}=\frac{3}{2}\frac{\hbar \omega }{\tau_{\mathrm{LO}}}\left(e^{\frac{\hbar \omega }{k_{B}T_{a}}}-e^{\frac{\hbar \omega }{k_{B}T_{C}}}\right)\frac{N_{\mathrm{LO}}\left(T_{a}\right)}{N_{\mathrm{LO}}\left(T_{C}\right)}{\left(\frac{k_{B}T_{C}}{\hbar \omega }\right)}^{2}e^{-\frac{\hbar \omega }{k_{B}T_{C}}}$$

where *ħω* is the LO phonon energy involved in HC cooling (∼9 meV, which corresponds to the stretching of the Pb─Br bonds [42]), *τ*_LO_ is the decay time of LO phonons, *T*_a_ is the acoustic phonon temperature, and *N*_LO_(*T*) is the occupation number of LO phonons at a specific temperature. The solid lines in Figure 3a suggest that *J*_r_ for varying densities initially follows the carrier‐phonon coupling model with different *T*_a_ (i.e., a higher <*N*_0_> results in a stronger heating of acoustic modes). However, *J*_r_ with higher carrier densities is greatly enhanced compared to the prediction of the carrier‐phonon model when the HC population cools below 700 K, indicating the existence of another slow HC cooling process.

In the case of CsPbBr_3_ NCs with ∼10 nm size, non‐radiative Auger recombination contributes significantly to carrier–carrier interactions for *n* larger than 10^18^ cm^−3^ [43, 44], leading to the heating of the electronic system (see Figure 3b). Such an Auger heating process, with a rate *γ*_A,_ competes dynamically against carrier‐phonon cooling, with the rate *γ*_ph_. This can be considered phenomenologically in the following way: *γ*_ph_ positively correlates with the carrier temperature, thus dominating the initial HC cooling with high *T*_C_; however, when *T*_C_ decreases to a level that *γ*_ph_ becomes smaller than *γ*_A_, Auger heating becomes the key process governing HC cooling. This nonequilibrium competition can accurately describe observations of the slow cooling process in Figure 3a, which only manifests at the middle *T*_C_ regime (i.e., *T*_C_ < 700 K) with high carrier densities. Meanwhile, from a quantitative perspective, Achermann et al. showed that *T*_C_ in the Auger heating regime is proportional to *γ*_A_ [37, 45]. The Auger decay of the *N* electron–hole pairs produces the (*N*−1) state (i.e., occurs in a bimolecular fashion) and thus, *T*_C_ can be approximately correlated to <*N*>*^2^*. This quadratic relationship is confirmed in Figure 3c, in which we compare the time‐resolved *T*_C_ to the squared bleaching dynamics (i.e., *N*_t_) of CsPbBr_3_ NCs. It can be seen that both transients show nearly identical behavior besides the initial phonon‐dominated cooling stage, confirming again the significant role of Auger heating in CsPbBr_3_ NCs. In contrast, contributions of Auger heating to the energy loss rate of SCs have been greatly suppressed, exhibiting a cooling trend nearly matching the carrier‐phonon coupling model. As shown in Figure 3d, *J*_r_ of SCs with high carrier densities reduces quickly by several orders of magnitude in the middle *T*_C_ regime (i.e., *T*_C_ < 700 K) until *T*_C_ approaches the lattice temperature, consistent with the observation in Figure 2c.

**FIGURE 4:**
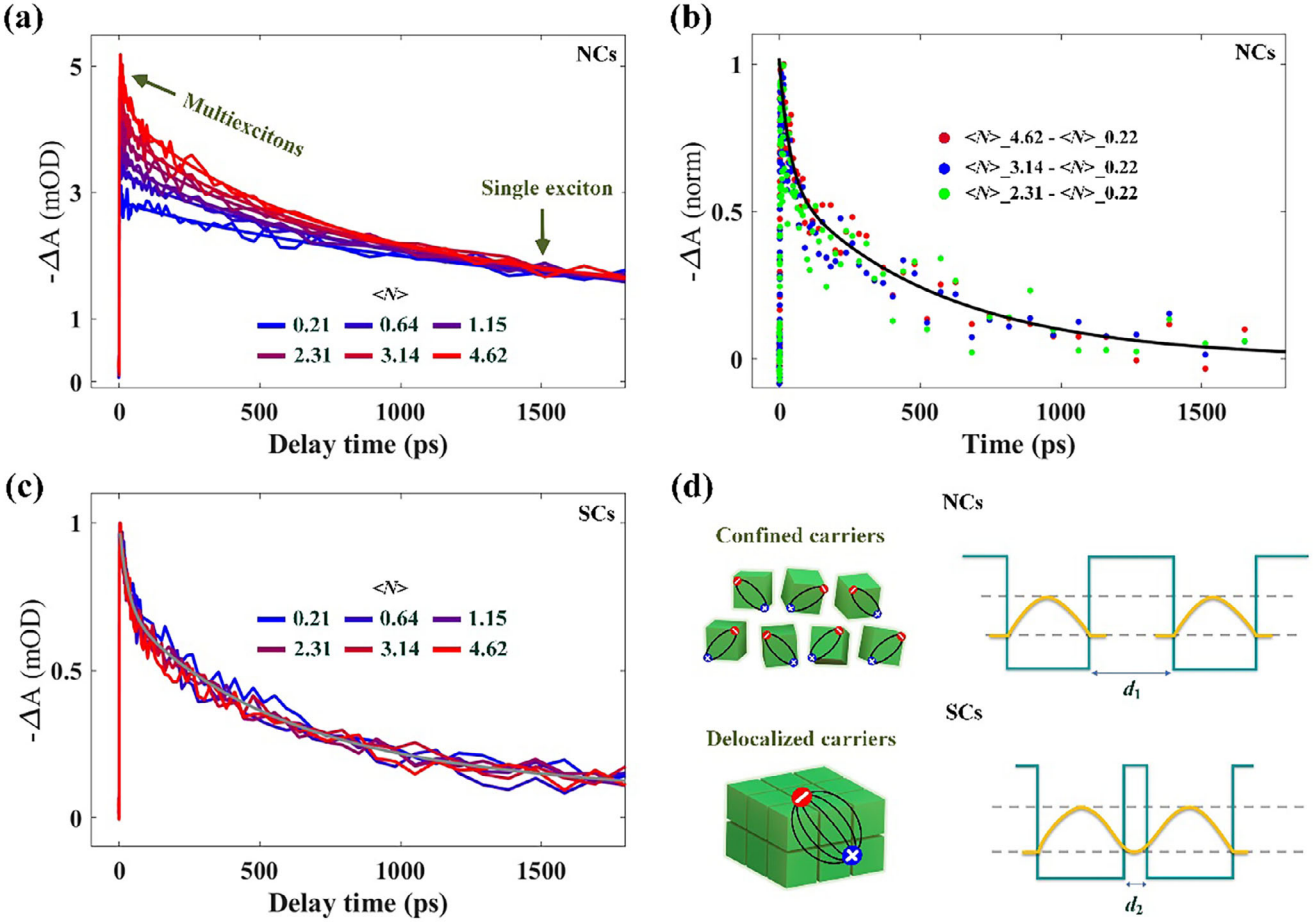
Characterizations of Auger dynamics in CsPbBr_3_ NCs and SCs. (a) Carrier density‐dependent PB kinetics of NCs excited at 400 nm. (b) The subtracted dynamics of PB kinetics in Figure 4a and their exponential fitting (the black curve). (c) Carrier density‐dependent PB kinetics of SCs excited at 400 nm. (d) Scheme illustration of different carrier–carrier interactions in SCs and NCs.

To verify the above analyses, carrier density‐dependent PB dynamics, reflecting the state‐filling effect of electrons and holes, are used to characterize the Auger lifetimes of NCs and SCs [46, 47]. The PB dynamics of NCs for <*N*> ranging from 0.21 to 4.62, normalized to the long‐lived tail corresponding to single excitons, are shown in Figure 4a. It can be found that with a low carrier density, the PB dynamics are dominated by a relatively slow, nearly single‐exponential decay trace. As <*N*> increases, fast decay components emerge with progressively larger amplitudes, manifesting the occurrence of many‐body recombination. Accordingly, such a high‐order process is deconvoluted using a well‐established subtraction procedure [48], which can be fitted to a bi‐exponential decay with time constants of ∼31 and ∼572 ps, respectively (see Figure 4b). It is worth mentioning that the fast timescale is consistent with the nonradiative biexciton Auger observed in CsPbBr_3_ NCs [47], while the slow recombination matches the biexciton radiative lifetime in CsPbBr_3_ NCs (i.e., ∼1 ns) [46]. As a comparison, the PB dynamics of NCs in Figure 4c are almost independent of <*N*> up to ∼4.62. This is in contrast to the fast decay component that emerges in NCs, suggesting a suppressed Auger effect. Based on the above results, we schematically illustrate in Figure 4d that as the NCs self‐assemble into superlattices, the enhanced nanocube coupling, arising from the shortened interparticle distances, leads to the spatial delocalization of electrons and holes [49, 50, 51]. As a result, the efficient carrier–carrier interactions caused by quantum confinement in NCs have been weakened, thus accounting for the reduction of Auger heating in CsPbBr_3_ SCs.

Regarding the potential influence of structural percolation (see Figure S2) on the measured optical response, we propose that the observed HC cooling dynamics are predominantly intrinsic to individual SL domains rather than being governed by inter‐SL interactions. This is supported by several considerations. First, the HC cooling in CsPbBr_3_ SLs occurs on an ultrafast timescale, which is orders of magnitude faster than the typical timescales for inter‐domain carrier diffusion or energy transfer (hundreds of picoseconds to nanoseconds), often limited by interfacial potential barriers. Second, the structural disorder and random orientations at the percolation necks effectively act as bottlenecks, suppressing long‐range electronic coherence and localizing the cooling process within each highly ordered domain [52]. Most importantly, spatially resolved TA measurements across multiple random locations (see Figures S9 and S10) reveal nearly identical 2D TA maps and *T*_c_ decay kinetics. This high degree of spatial uniformity provides direct experimental evidence that the measured dynamics are robust and independent of local connectivity or percolation density, confirming they represent the intrinsic photophysical properties of the CsPbBr_3_ SLs.

## 3 Conclusions

In this work, we have demonstrated that long‐range electronic coupling in synthesis‐assembled CsPbBr_3_ SCs enables rapid HC cooling dynamics across low (2 × 10^17^ cm^−3^) and high (4.3 × 10^18^ cm^−3^) excited carrier regimes. At low carrier densities, the SCs intrinsically enhance the electronic density of states through cooperative interactions, accelerating HC cooling by a factor of 2 compared to isolated nanocubes. Meanwhile, at the high carrier densities, carrier delocalization in the SCs suppresses Auger‐dominated heating, resulting in a faster HC cooling by over an order of magnitude. Our results suggest that the self‐assembly concept avoids trade‐offs inherent to conventional strategies, preserving intrinsic material properties, while decoupling HC dynamics from competing photophysical pathways. Although conventionally, the presence of long‐chain dielectric ligands on nanocrystal surfaces may limit the active layer thickness in devices by suppressing carrier transport, the development of SLs and self‐assembled nanostructures offers a robust pathway to overcome these traditional performance bottlenecks. Benefiting from their highly ordered structural coherence, SLs not only significantly enhance photon out‐coupling efficiency via aligned transition dipoles, but also improve carrier transport uniformity and overall device stability by minimizing energetic disorder [53, 54, 55, 56]. Crucially, our present work further reveals that the enhanced electronic coupling intrinsic to SLs can markedly accelerate the hot carrier cooling rate. This effective regulation of ultrafast carrier dynamics provides critical mechanistic insights, underscoring the immense potential of integrating SLs into next‐generation high‐performance optoelectronic applications, particularly for the development of high‐efficiency, low‐droop LEDs and low‐threshold lasers.

## Author Contributions

**Zhenzhong Lian**: investigation, validation. **Yadong Han**: investigation, validation, methodology. **Junhong Yu**: conceptualization, investigation, validation, writing – review and editing, writing – original draft. **Manoj Sharma**: investigation, methodology, validation, writing – review and editing. **Chang Cao**: investigation, validation. **Jacek J. Jasieniak**: writing – review and editing, resources, supervision. **Baiquan Liu**: investigation, validation, resources, supervision. **Naufan Nurrosyid**: investigation, validation. **Lan Nguyen**: investigation, validation. **Ke Wang**: investigation, validation.

## Conflicts of Interest

The authors declare no conflict of interest.

## Supporting information




**Supporting File**: advs75913‐sup‐0001‐SuppMat.docx.

## Data Availability

The data that support the findings of this study are available from the corresponding author upon reasonable request.
