# Supplementary material for: Ultrafast Hot‐Carrier Cooling in CsPbBr3 Supercrystals via Long‐Range Electronic Coupling
Source: Adv Sci (Weinh). 2026 Jun 9:e75913. Online ahead of print. doi: 10.1002/advs.75913 (PMC13336793; doi:10.1002/advs.75913)
Supplement: Supplementary file 1 — Supporting File: advs75913‐sup‐0001‐SuppMat.docx. [file ADVS-9999-e75913-s001.docx]

**SUPPLEMENTARY INFORMATION**

**Ultrafast hot-carrier cooling in CsPbBr_3_ supercrystals via long-range electronic coupling**

Junhong Yu,^1,#,*^ Manoj Sharma,^2, #,*^ Yadong Han,^1^ Ke Wang,^1^ Zhenzhong Lian,^1^ Chang Cao,^2^ Lan Nguyen,^2^ Naufan Nurrosyid,^2^ Baiquan Liu,^3,*^ Jacek J. Jasieniak,^2^

*^1^College of Physics and Electronic Engineering, Chongqing Normal University, Chongqing, 401331, China*

*^2^Department of Materials Science and Engineering, Monash University, Clayton Campus, Melbourne, Victoria 3800, Australia*

*^3^School of Electronics and Information Technology, Sun Yat-sen University, Guangzhou 510275, China*

^#^ *Contributed equally.*

*^*^To whom correspondence should be addressed. Email: jyu012@e.ntu.edu.sg (JY); manoj.sharma@monash.edu (MS);* *liubq33@mail.sysu.edu.cn (BL).*

# Supplementary Figure 1


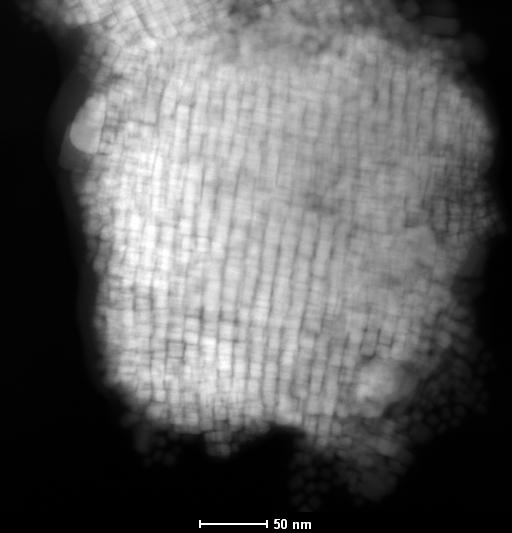


**Figure S1. HAADF-STEM images of SCs with the scale bar of 50 nm.**

# Supplementary Figure 2


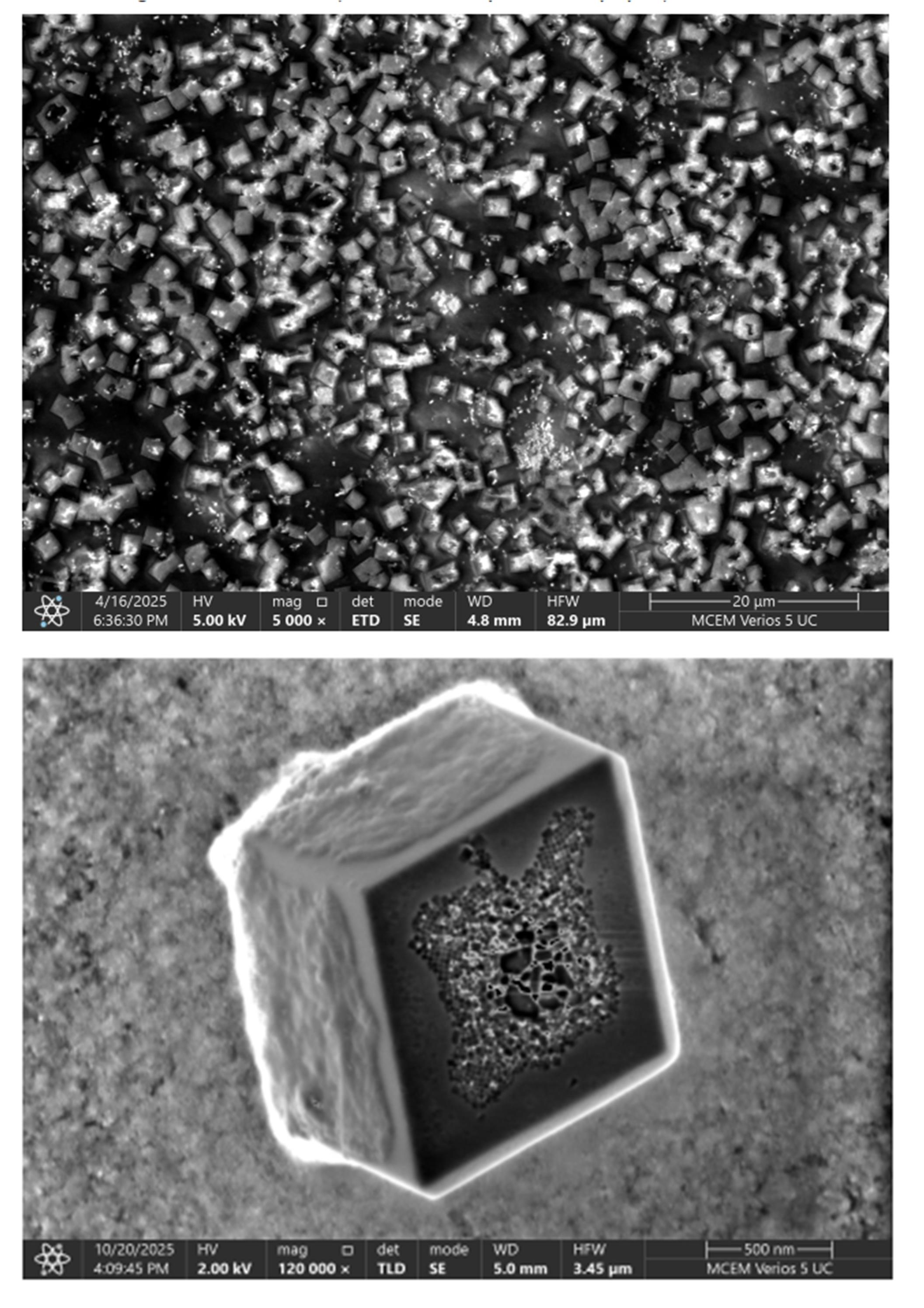


**Figure S2. SEM images of SCs with different mgnifications.**

# Supplementary Figure 3


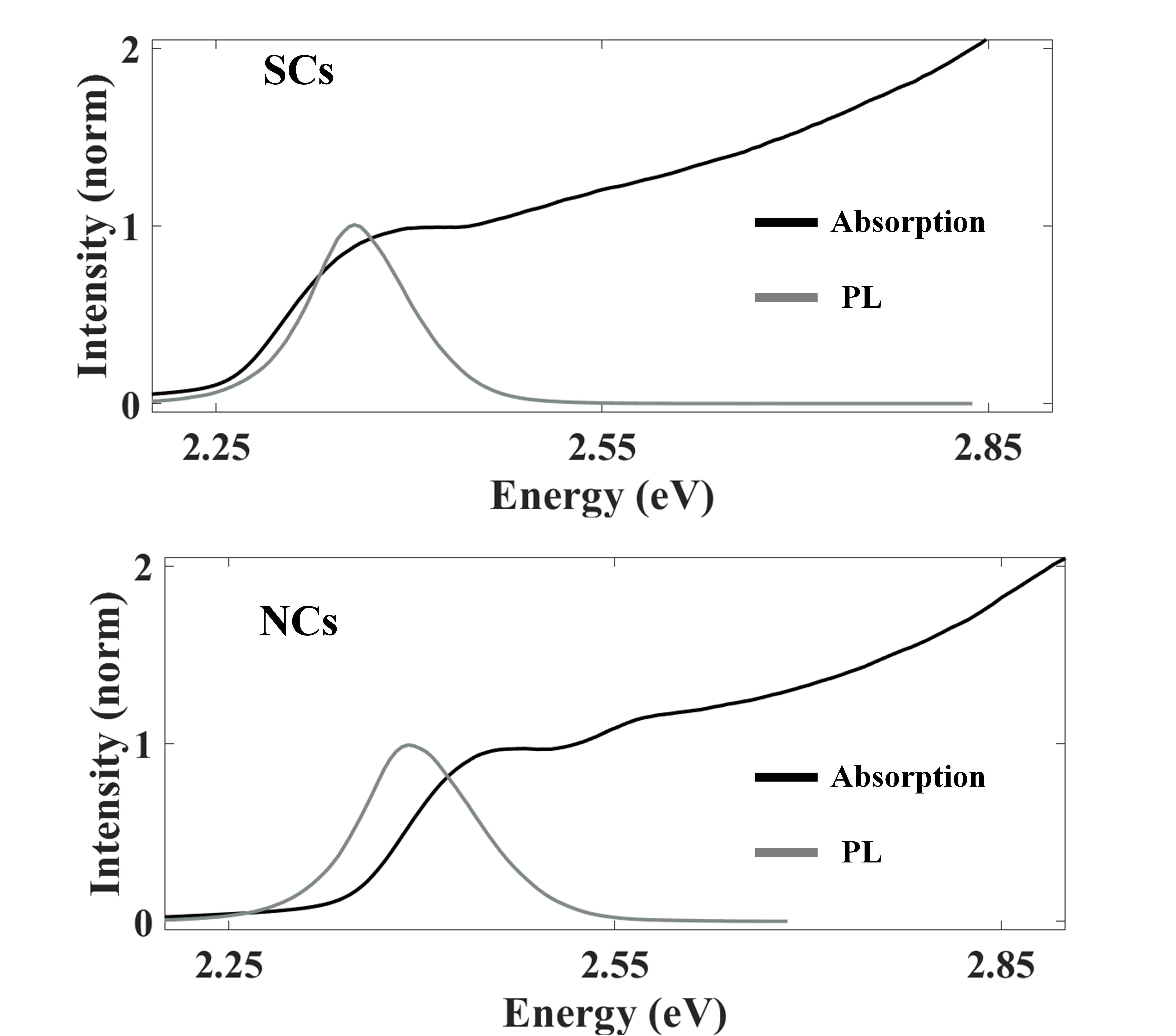


**Figure S3. Upper: Absorption (black) and PL spectra (grey) of colloidal SCs; Lower: Absorption (black) and PL spectra (grey) of colloidal NCs.**

# Supplementary Figure 4


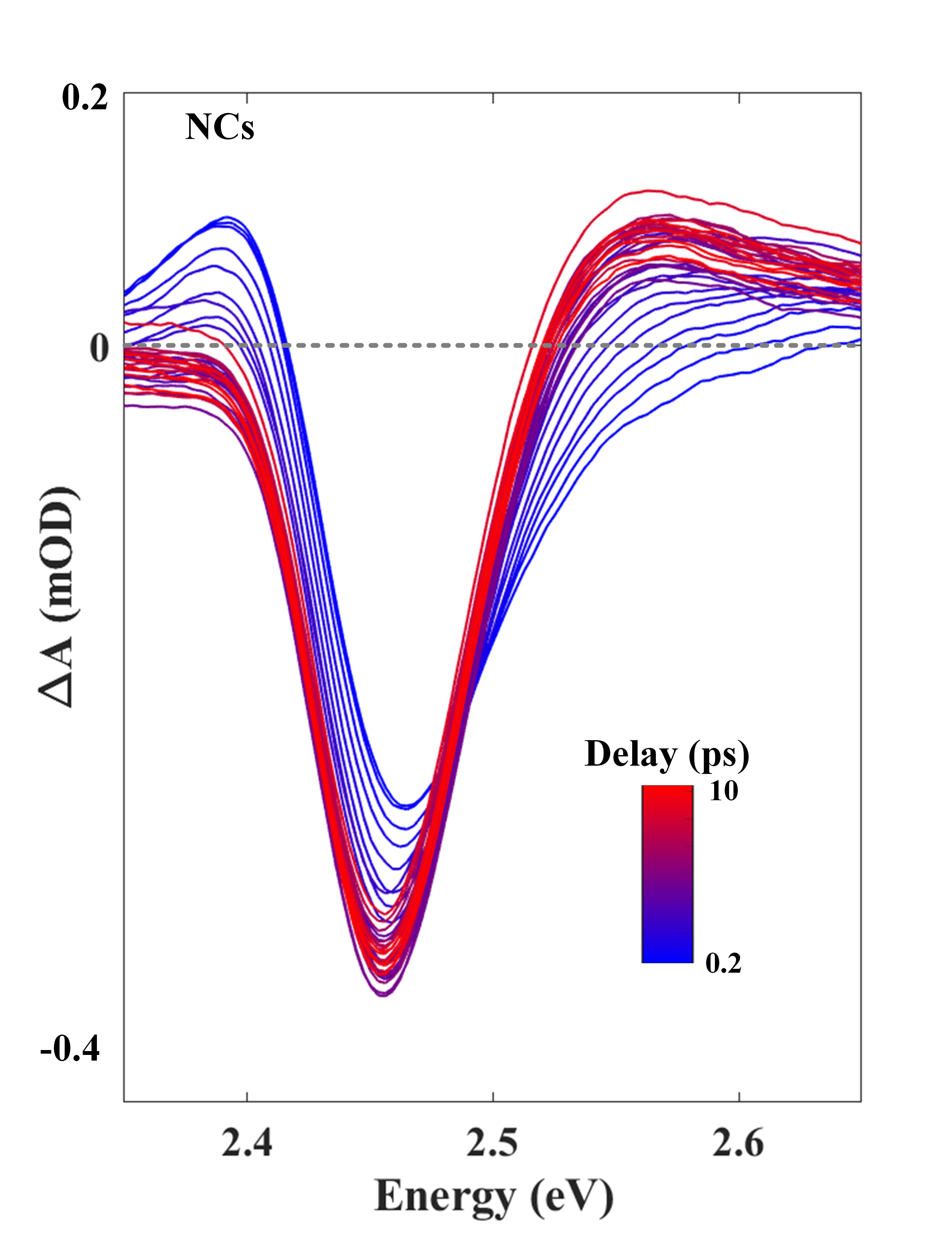


**Figure S4. Delay time dependent TA spectra of CsPbBr_3_ NCs with a carrier density of ~2.1×10^17^ cm^-3^.**

# Supplementary Figure 5


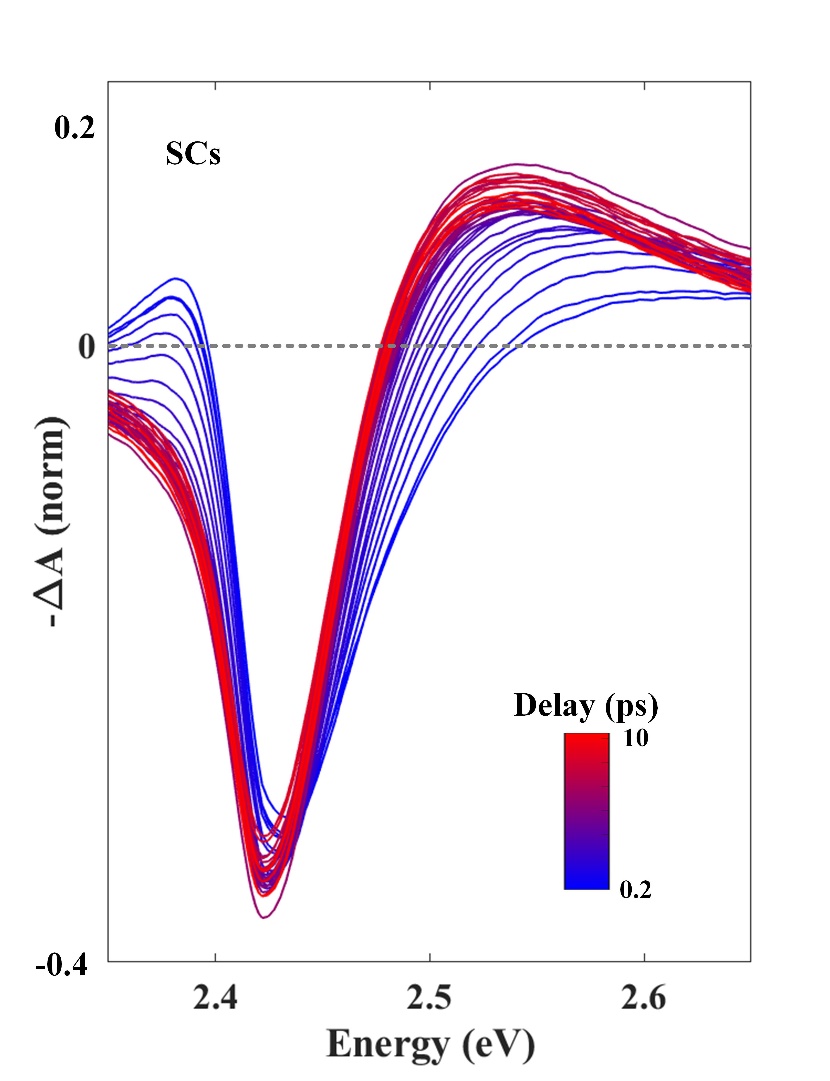


**Figure S5. Delay time dependent TA spectra of CsPbBr_3_ SCs with a carrier density of ~2.1×10^17^ cm^-3^.**

# Supplementary Figure 6


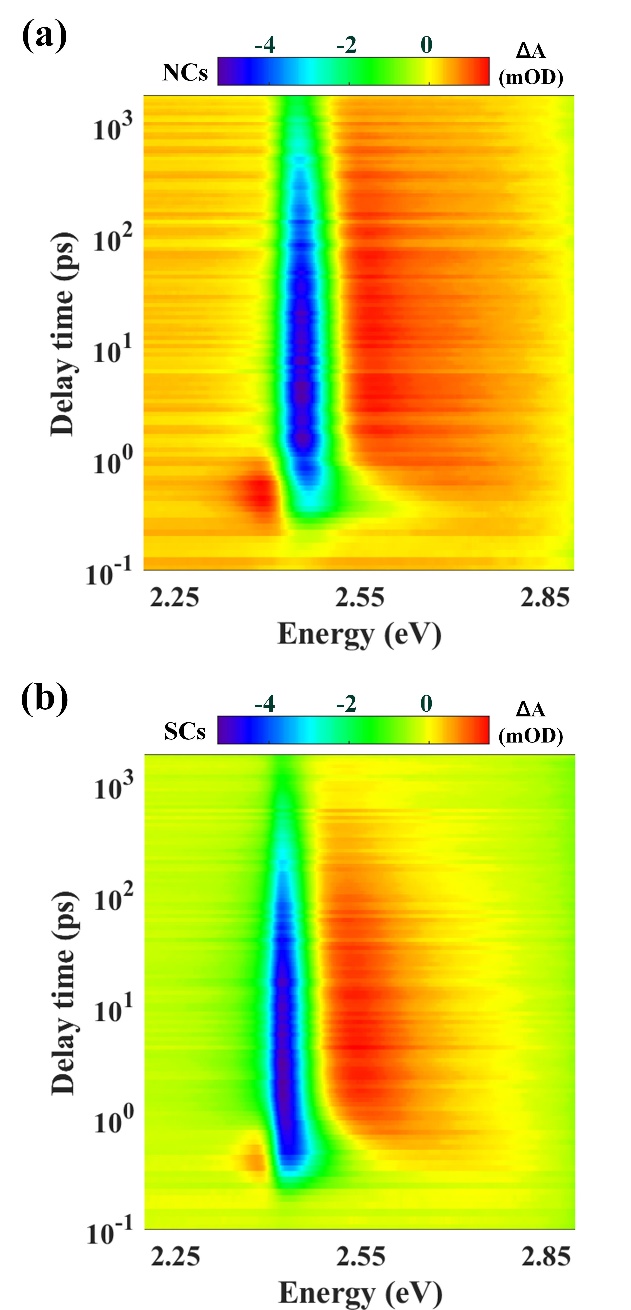


**Figure S6. Pseudocolor TA spectroscopies of CsPbBr_3_ NCs (a) and SCs (b) with *n* = ~2.31×10^18^ cm^-3^.**

# Supplementary Figure 7


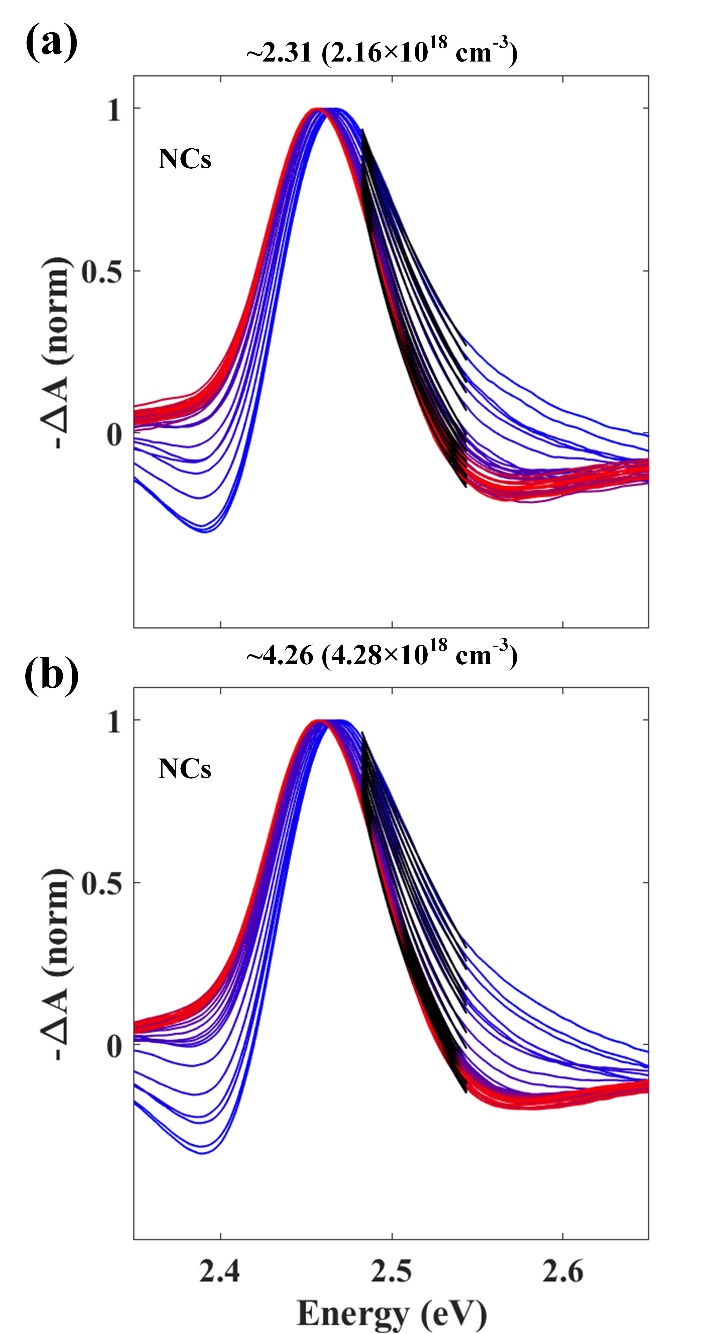


**Figure S7. Normalized TA spectra of NCs wit**h ***n* = ~2.31×10^18^ cm^-3^ (a) and *n* = ~4.62×10^18^ cm^-3^ (b). The** b**lack curves mark the data used for extracting the carrier temperature.**

# Supplementary Figure 8


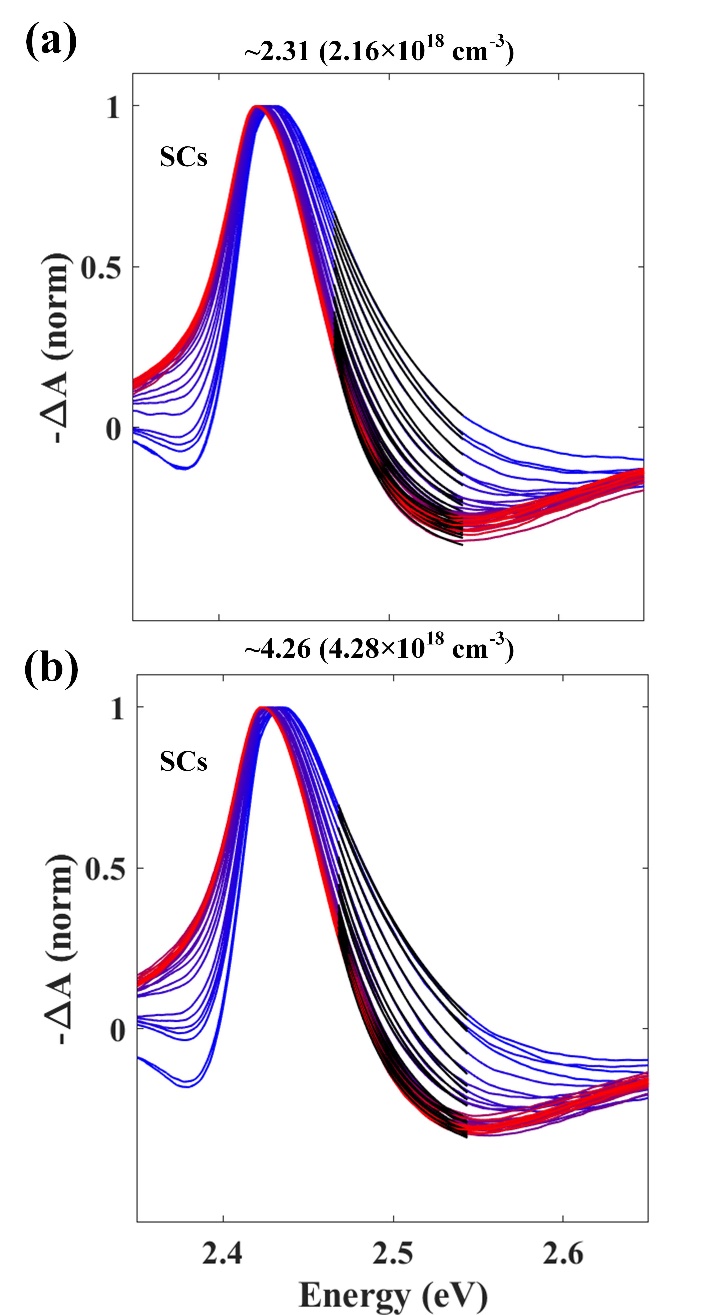


**Figure S8. Normalized TA spectra of SCs wit**h ***n* = ~2.31×10^18^ cm^-3^ (a) and *n* = ~4.62×10^18^ cm^-3^ (b). The** b**lack curves mark the data used for extracting the carrier temperature.**

**Supplementary Figure 9**


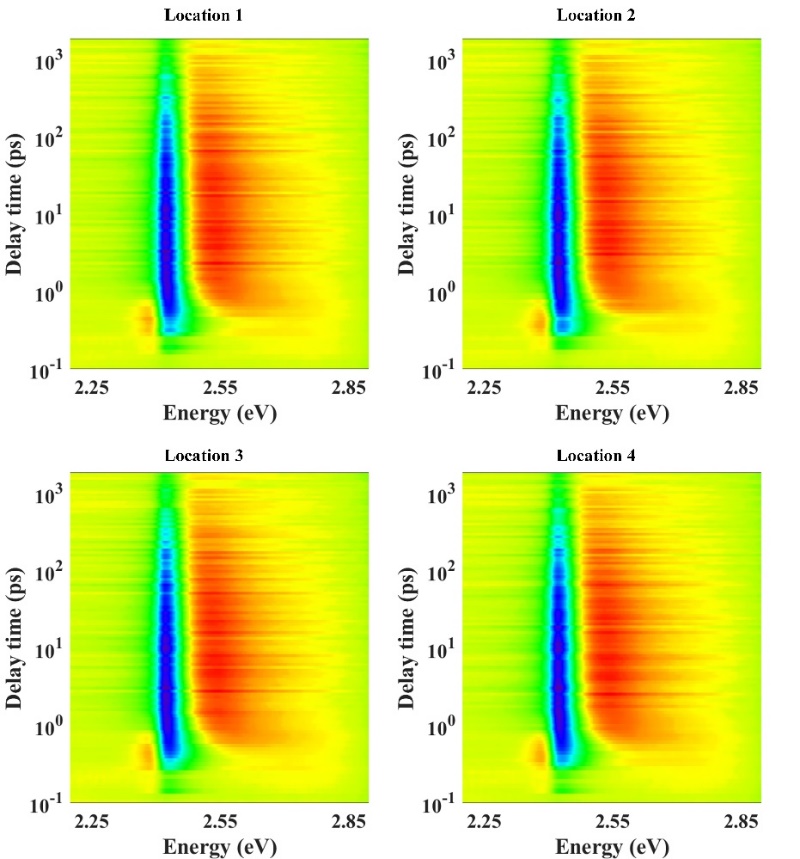


**Figure S9.**  **Pseudocolor TA spectroscopies of CsPbBr_3_ SCs collected at different locations of the thin film with low excitation densities (*n* = ~2.1×10^17^ cm^-3^) and a photoexcitation energy of 3.1 eV.**

**Supplementary Figure 10**


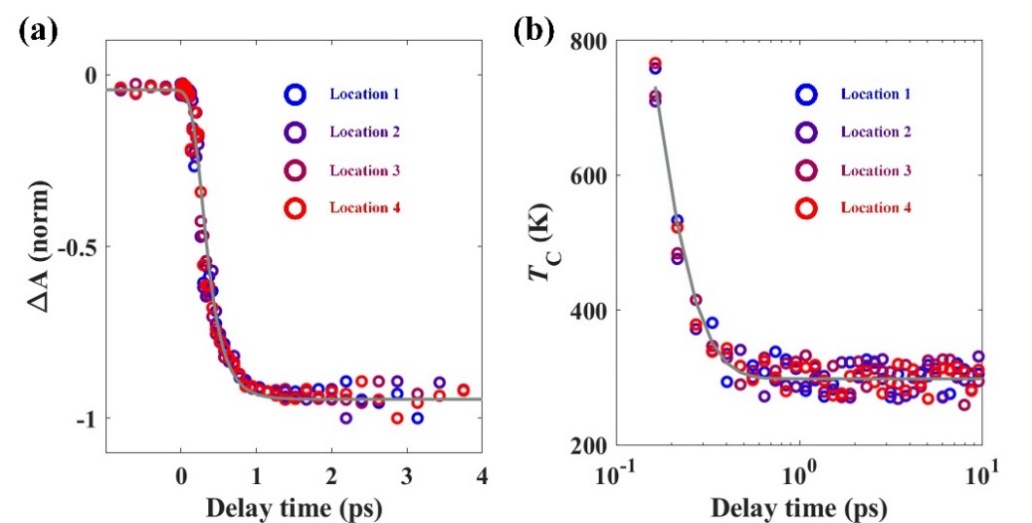


**Figure S10.**  **Corresponding analysis based on the TA data presented in Fig. S9. (a) Early time TA dynamics of SCs probed at the positions of PB features. (b) Hot-carrier temperature of SCs as a function of delay times.**

**Supplementary Figure 11**


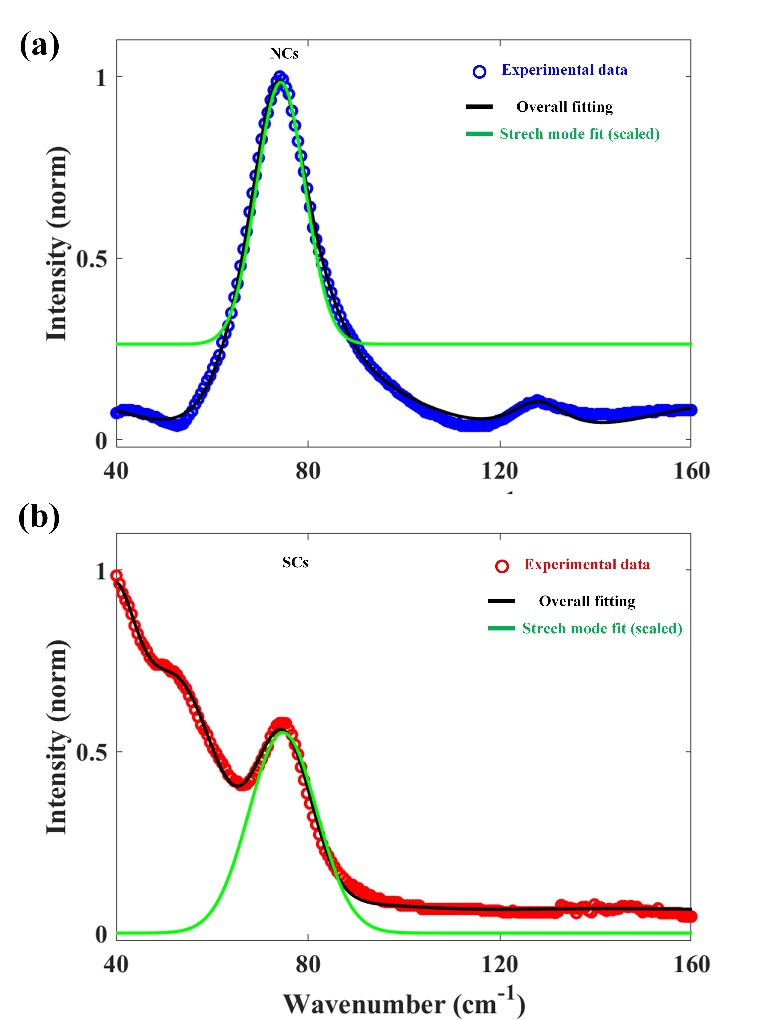


**Figure S11. Raman spectra of CsPbBr3 NCs (a) and SCs (b).** The spectra reveal that the Pb-Br stretching mode (located at 74 cm^-1^), which dictates hot carrier energy loss, exhibits peak broadening in SCs compared to isolated NCs (i.e., 17.4 cm^-1^ versus 10.1 cm^-1^). Based on the energy-time uncertainty principle, such spectral broadening directly signifies a faster phonon decay rate and a correspondingly shorter LO-phonon lifetime.

**Supplemental Note 1**

**Transient absorption measurements.** A 1 kHz regenerative amplifier with an 800 nm output wavelength and a 100 fs pulse width, which is seeded by the 80 MHz mode-locked Ti-sapphire oscillators, was used to pump the produce optical parametric amplifier and generate the pump pulse with the maximum energy up to mJ. To generate the white light continuum probe beam spanning from 400 to 1500 nm, we have split a small portion of the fundamental 800 nm pulse from the regenerative amplifier with an energy of ~10 μJ and then incident on sapphire crystals with different thickness for the continuum probe beam generations. A commercial spectrometer was used to collect the probe beam spectra in the ultraviolet-visible region with and without the pump excitations.
